# Statistical Approach for Improving Genomic Prediction Accuracy through Efficient Diagnostic Measure of Influential Observation

**DOI:** 10.1038/s41598-020-65323-3

**Published:** 2020-05-21

**Authors:** Neeraj Budhlakoti, Anil Rai, D. C. Mishra

**Affiliations:** Centre for Agricultural Bioinformatics, ICAR-Indian Agricultural Statistics Research Institute, 110012 New Delhi, India

**Keywords:** Computational biology and bioinformatics, Statistical methods

## Abstract

It is expected the predictive performance of genomic prediction methods may be adversely affected in the presence of outliers. In agriculture science an outlier may arise due to wrong data imputation, outlying response, and in a series of trials over the time or location. Although several statistical procedures are already there in literature for identification of outlier but identification of true outlier is still a challenge especially in case of high dimensional genomic data. Here we have proposed an efficient approach for detecting outlier in high dimensional genomic data, our approach is p-value based combination methods to produce single p-value for detecting the outliers. Robustness of our approach has been tested using simulated data through the evaluation measures like precision, recall etc. It has been observed that significant improvement in the performance of genomic prediction has been obtained by detecting the outliers and handling them accordingly through our proposed approach using real data.

## Introduction

Genomic selection (GS) has been a popular choice for selection of appropriate candidates for breeding in the current research arena of plant and animal science. Various studies has been carried out in recent past. GS is an advance method of breeding where genome-wide dense markers information is used to predict genetic merit of an individuals in a breeding programme. In today’s scenario GS is a promising tool for improving genetic gain of individuals under study. Genomic selection is firstly introduced by Meuwissen *et al*.^1^. In this approach individual effect of each marker is estimated and sum of all markers effect is used for calculation of genotypic value i.e. Genome Estimated Breeding Value (GEBVs) of each individual.

GS process starts with building a statistical model from individuals having both genotypic and phenotypic data (i.e. training set), this model is further used for estimation of GEBVs for individuals having only genotypic information. Individuals are then ranked on the basis of GEBVs and subsequently superior individuals are selected. Genomic selection methods have been successfully applied for plants^2,3^ and animals^4–7^. However, success of genomic selection depends on the quality of the data suitable for implementing the various statistical models. But in practical situation genomic data quality seldom fulfill the ideal condition and often having many constraints such as presence of influential observations, missing points, noise etc.

Influential observations can potentially have devastating effects on genome estimated breeding values^8^. These influential observations can be the results of wrong data imputation, outlying response, and in a series of trials over the time or location. Detection of influential observation has been an extensive research area based on linear regression approach^9–12^. Some of most widely used measures for this are Cook’s D, DFBETA, DFFITS, Atkinson’s Ci, COVRATIOi. Among them Cook’s D is one of the most commonly used measure for outlier detection through linear regression technique^10^. Various statistical model with t-distributed error has been proposed (Bayesian with t-linear model^13^, Gaussian process with t-likelihood^14^, Regression with t-error^15^) as robust method against treating the outlier. Lange *et al*.^15^ have applied this model (Regression with t-error) to various datasets and concluded that it can handle outliers and address robustness concerns practically and routinely in a wide range of settings. However, discriminating true outlier from non-outlier is still a challenge especially in case of high dimensional genomic data. The key complication in handling the problem of outlier is that distinguishing mild outlier from regular observations and masking of true outlier^16^. In high dimensional genomic data, where no of markers (p) are greater than no of individuals (n) creates a problem termed as large p small n problem (p > n). This is a very common phenomena in genomics and molecular biology research now a days. In such cases, penalized regression based approach such as Least Absolute Shrinkage and Selection Operator (LASSO) could be a preferable choice as it takes care of n ≪ p problem by shrinking the estimates of some less significant markers and dropping others from the model. Increased use of LASSO has been motivated by plenty of high dimensional biological data. But it becomes very crucial when some influential observations are present in high dimensional genomic data as each observation has tremendous effect on model selection and interpretation. So it is quite imperative to examine effect of influential observation before implementing the LASSO regression. Hence new measure for detection of influential observation in high dimensional genomic data is a need of hour for improving GEBV’s.

Rajaratnam *et al*.^17^ recently developed approach for outlier detection for high dimensional data by considering the LASSO regression technique. In their approach they have proposed four measures i.e. df-model, df-lambda, df-regpath and df-cvpath for detection of influential observations influenced by different aspect of LASSO regression directly or indirectly. However, the results coming from these measures are not consistent i.e. different influential points are detected from these measures. In order to produce more concrete and consistent results, a meta-analysis based approach can be applied where an improved measure of outlier detection can be developed based on integration of these measures using p-values^18–20^.

In this study, an improved measure for detection of influential observation has been developed using above mentioned approach. Performance of the developed measure has been empirically evaluated and it was observed that the outliers detected from this measure are more accurate. This developed method has been implemented in the case of genomic selection data (real and simulated) and results shows that there is remarkable improvement in the prediction accuracy of GEBVs.

## Material and Methods

LASSO was first time introduced by Tibshirani^21^. LASSO minimizes the sum of squares of residuals subject to a constraint on sum of absolute values of regression coefficients. It is different from usual regression as it adds some additional penalty to usual regression estimator. So it diminishes the effect of less important βs (i.e. marker effect) and reduces least important βs as zero.

Statistical formulation of LASSO estimates can be defined as:

1 $${\hat{\beta }}^{lasso}=\begin{array}{c}argmin\\ \beta \end{array}\mathop{\sum }\limits_{i=1}^{n}{({y}_{i}-\mathop{\sum }\limits_{j=1}^{p}{x}_{ij}{\beta }_{j})}^{2}$$

subject to ${\sum }_{j=1}^{p}|{\beta }_{j}|\le t$, $i=1,\ldots ,n$ (individuals), $j=1,\ldots ,p$ (markers), ${Y}_{i}$ is the phenotypic value for individual $i$, ${x}_{ij}$ is an element of the incidence matrix corresponding to individual $i$ and marker $j$, ${\beta }_{j}$ is the marker effect for marker $j$. It has been assumed that response variable has zero mean. The constraints ${\sum }_{j=1}^{p}|{\beta }_{j}|\le t$ shrinks effects of variables and sets some of them to zero.

We can also write the LASSO problem in the equivalent Lagrangian form:

2 $${\hat{\beta }}^{lasso}=\begin{array}{c}argmin\\ \beta \end{array}\left\{\frac{1}{2}\mathop{\sum }\limits_{i=1}^{n}{({y}_{i}-\mathop{\sum }\limits_{j=1}^{p}{x}_{ij}{\beta }_{j})}^{2}+\lambda \mathop{\sum }\limits_{j=1}^{p}|{\beta }_{j}|\right\}$$

Here ${\sum }_{j=1}^{p}|{\beta }_{j}|$ is ${l}_{1}$ norm penalty on $\beta$ which results in sparsity of solution and λ is a regularization parameter. Computing the LASSO solution is quadratic programming problem which can be obtained through efficient algorithm like Least Angle Regression (LARS)^22^. Other important question to be addressed in this case is calculation of upper limit of sum of absolute value of predictor variable, for this cross validation approach can be used^23^.

Here we have used a recently proposed approach for detection of influential observation based on LASSO technique^17^. They proposed four different measure i.e. df-model- it measure the change in model selected; df-lambda: it measure the change in *λ*, where *λ* is a regularization parameter in LASSO regression path, df-regpath: it measure the changes observed in LASSO regularization path and df-cvpath which observe changes in LASSO cross-validation path. These measures detects outlier from high dimensional genomic data based on LASSO regression. It can be observed that all these measures i.e. df-model, df-lambda, df-regpath and df-cvpath detects influential observations which affects model directly or indirectly, has difference in their results regarding detection of influential observation, it means that there is lack of concordance among them. In order to overcome this limitation, we have proposed a more robust measure for detection of influential observation by integrating above discussed measure using p-values based meta-analysis approach.

### Approach of proposed measure

In order to develop a robust statistics for detection of influential measure, we have used p-value based meta-analysis approach. In this approach, we have combined the above mentioned four measures on the basis of their p-values. We used various methods for combining these p-values and explored the performance of each method. The brief description of this approach has been as follows. Let’s say, there are *K* independent test and their corresponding *p*-values are *p*_1_, *p*_2_,…, *p*_*K*_. Under H_0_, it is assumed that *p*-values from different methods (for individual observations) are uniformly distributed between 0 and 1 (i.e. *p*_*k*_ ~ U [0, 1]). To get overall statistical significance for the hypothesis under test (H_0_ i.e. null hypothesis vs. H_1_ alternative hypothesis), individual p-values for each observation/genotype from different methods (i.e. df-model, df-lambda, df-regpath and df-cvpath) can be combined. Methods used for this purpose has been summarized in Table 1.

**Table 1:** List of methods used in study for combining p-value to calculate overall significance.

Methods	Test Statistic	Transformed Variable	Dist. under H_0_	Reference
Inverse Chi-Square/Fisher	$L=\mathop{\sum }\limits_{k=1}^{K}{Z}_{k}$	*Z*_*k*_ = −2log*p*_*k*_	${\chi }_{2K}^{2}$	^20,39^
Logit/Pearson	$S=\mathop{\sum }\limits_{k=1}^{K}{S}_{k}$	${S}_{k}=log[{p}_{k}/(1-{p}_{k})]$	${t}_{5K+4}$	^40^
Meanp	$W=(0.5-\bar{p})\sqrt{12k}$	$\bar{p}=\mathop{\sum }\limits_{k=1}^{K}{p}_{k}/K$	N(0, 1)	^19^
Sumz/Stouffer’s method	$Z=\frac{{\sum }_{k=1}^{K}{w}_{k}z({p}_{k})}{\sqrt{{\sum }_{k=1}^{K}{w}_{k}^{2}}}$	NA	N(0, 1)	^41^

where *p*_*k*_: Statistical significance value from k^th^ methods for a individual or genotype; K: Different methods for which p-values to be combined; df: degrees of freedom; N(): Normal distribution; t: Central t-distribution; χ²: Central Chi-square-distribution.

Using this approach (Table 1), the final statistical significance value i.e. combined p-values for selected observation/genotype has been calculated and influential observation is identified based on suitable p-value cut-off. Source code for our proposed approach can also be accessed from github repository at https://github.com/BudhlakotiN/OGS.

### Experimental dataset

In order to check the robustness of our approach the same has been validated using real data. We have used total six datasets in the current study. Detailed discussion regarding each of dataset is given below.

### Dataset 1: Wheat

Wheat lines were genotyped using 1447 Diversity Array Technology markers generated by Triticarte Pty. Ltd. (Canberra, Australia; http://www.triticarte.com.au). These markers may take two different values i.e. their presence (1) or absence (0). This data set includes 599 lines observed for trait grain yield (GY) for four mega environments. However for our convenience we have just considered GY for first mega environment. The final number of DArT markers after edition was 1279 hence same has been used in this study. Same has been also used in genomic prediction study^24,25^.

### Dataset 2: Maize

Maize dataset is generated by CIMMYT’s Global Maize Program^24^. It originally include 300 maize line with 1148 SNP markers. For marker with highest frequency is coded as 0 and lowest frequency as 1. Here trait under study is also GY, evaluated under draught and watered conditions. The average minor allele frequency in these data sets was 0.20. After some editing 264 maize lines with 1135 SNPs markers were available for final study^24^.

### Dataset 3–6: Wheat

This wheat dataset is generated from CIMMYT semiarid wheat breeding program which is comprised of 254 advanced wheat breeding lines genotyped for 1726 DArt markers^26^. This dataset is recorded for four phenotypic traits i.e. Days to heading (DTH), Thousand Kernel Weight (TKW), Yield (under irrigated condition hence denoted as Y_I_), Yield (under draught condition i.e. Y_D_). For convenience, here trait DTH is considered as Dataset-3, trait TKW as Dataset-4, trait Y_I_ as Dataset-5 and trait Y_D_ as Dataset-6.

### Simulation

**Figure 1:**
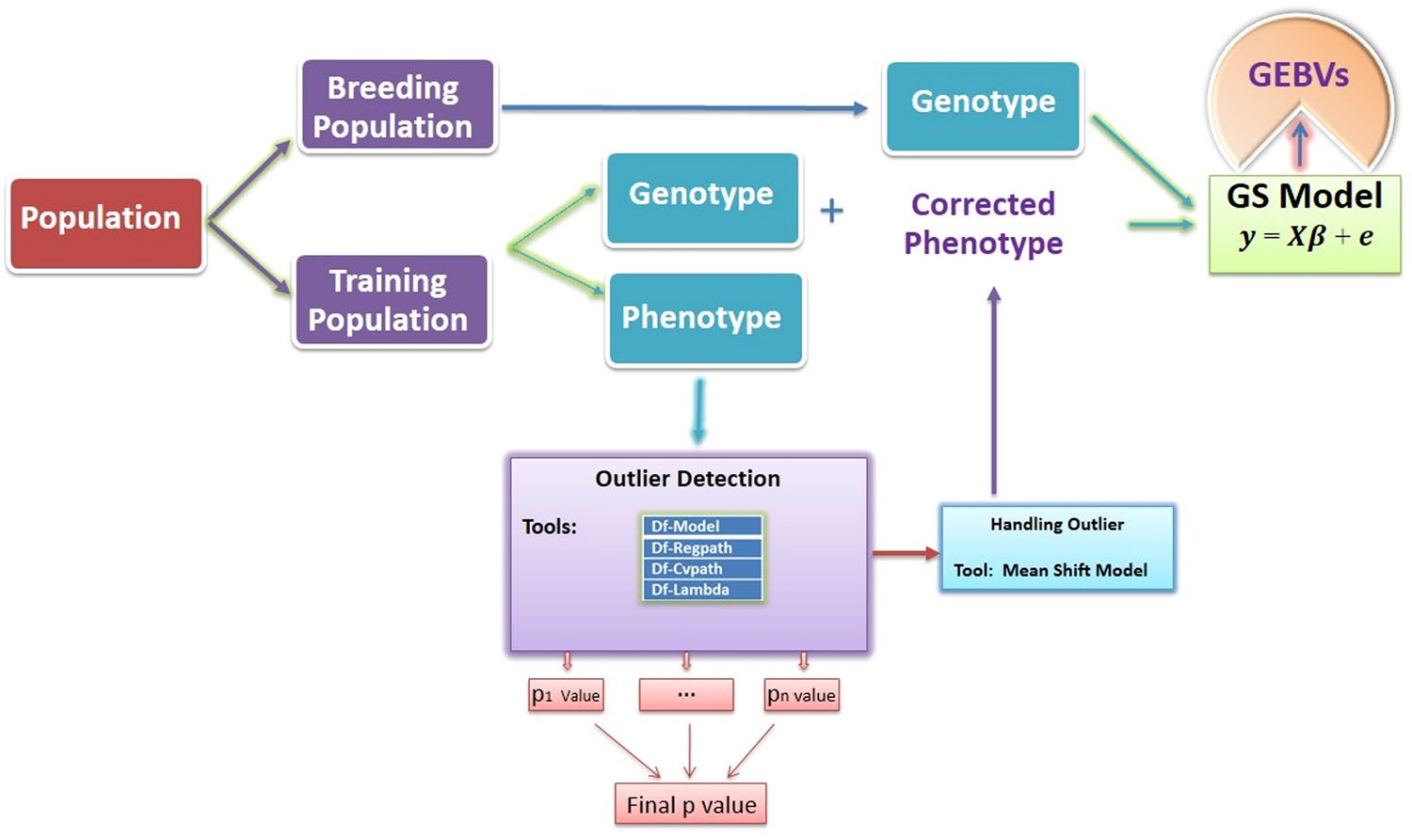
Operational workflow of the whole procedure used in the study.

For illustration simulated data were generated using QTL Bayesian interval mapping (“qtlbim”)^27^, a R based (R Development Core Team 2019) package. R is available at http://www.r-project.org and qtlbim package can be loaded from R library. This package has been used in various studies for simulation of data related to genomic selection^28–30^. The qtlbim package uses Cockerham’s model as the underlying genetic model. We have simulated a total of three data sets for genotypic and phenotype information. Here we have created range of diversified genetic architecture i.e. with very low heritability 0.10 to medium 0.5 and high heritability 0.7. Accordingly, we have simulated data at these particular heritability levels. For each stage we have simulated data for 1000 SNPs for 200 individuals. Simulated data have 10 chromosomes with 100 SNPs in each with specified length. Total 1000 markers are distributed over the all 10 chromosomes in such a way that each marker is equi-spaced over the chromosome. We have simulated normally distributed phenotype, with further no genotype or phenotype information missing. In order to check the sensitivity of all methods to detect true outlier, we have replaced 5% of observation and made them outlier (i.e. beyond mean ± 3*SD). Overview of whole workflow of the current study presented in Fig. 1.

#### Evaluation measure

As an evaluation measure, prediction accuracy and prediction error were used. Prediction accuracy can be defined as Pearson correlation coefficient (r) between observed phenotypic value and predicted phenotypic value.

If $\,\hat{Y}=X\hat{\beta }$, where $\hat{Y}$ is estimated response and $\hat{\beta }$ is estimated value of $\beta$, then correlation coefficient (r) can be expressed in following form:

3 $$r=\frac{{S}_{Y,\hat{Y}}}{{S}_{Y}{S}_{\hat{Y}}}$$

where $\,{S}_{Y,\hat{Y}}$ denotes the covariance between observed and predicted phenotypic value, ${S}_{Y}$ is standard deviation of observed phenotype and ${S}_{\hat{Y}}$ denotes standard deviation of predicted phenotype. Prediction Error (PE) can be simply defined as mean sum of square error (MSE) between observed phenotypic value and predicted phenotypic value. Same can be expressed using following formula (Eq. 3).

4 $$PE/MSE=\frac{1}{{n}_{test}}\mathop{\sum }\limits_{i=1}^{{n}_{test}}{({Y}_{i}-{\hat{Y}}_{i})}^{2}$$

Where ${Y}_{i}$ is observed response; ${\hat{Y}}_{i}$ is predicted phenotype value. It can be understood that $n$ is the total no. of individual’s i.e. $\,n={n}_{train}+{n}_{test}$, here $\,{n}_{train}$ denotes no of individuals in the training set and ${n}_{test}$ is no. of individuals in test set.

In order to assess performance of methods to identify true outlier (observation with added noise) and non-outlier (observation without any noise), we have used precision (i.e. proportion of True Positive (TP) to total positives (i.e. sum total of true positive (TP) and False Negative (FN), Eq. 5), recall (i.e. proportion of TP to TP and False Negative (FN), Eq. 6) and F1 score (i.e. harmonic mean of precision and recall, Eq. 7). All these can be computed using the following expressions:

5 $$\text{Precision}\,=\,\frac{\text{TP}}{\text{TP}+\text{FP}}$$

6 $$\text{Recall}\,=\,\frac{\text{TP}}{\text{TP}+\text{FN}}$$

7 $$\text{F}1=\sqrt{\text{Precision}\times \text{Recall}}$$

To calculate the overall performance of different methods, Technique for Order of Preference by Similarity to Ideal Solution (TOPSIS) has been used. It is a multi-criteria based decision making method given by Hwang and Yoon^31^. It is based on the impression that the selected alternative should have the shortest geometric distance from the positive ideal solution (PIS) and the longest geometric distance from the negative ideal solution (NIS)^32^. TOPSIS compares set of alternatives by giving some weights to each criteria followed by normalization of each single criteria and calculates the geometric distance between each alternative and ideal alternative. TOPSIS is based on the assumption of that criteria are monotonically increasing or decreasing. Here final rank has been calculated using R package ‘topsis’ motivated from TOPSIS method.

## Results and Discussion

Performance of our proposed method that how well it distinguishes true outlier from non-outlier, various measures like precision, recall and F1 score has been calculated for different datasets (generated at different heritability h^2^ = 0.1, 0.5 and 0.7) and presented in Table 2. It suggest that our proposed approach i.e. based on combining p-value outperformed in almost every scenario.

**Table 2:** Performance of different methods (in terms of Precision, Recall and F1 score) for different simulated datasets.

Heritability	Methods	Precision	Recall	F1
0.1	Df-Model	0.55	0.6	0.57
	Df-Regpath	0.875	0.7	0.78
	Df-Cvpath	0.85	0.6	0.71
	Df-lambda	0.46	0.6	0.52
	sumz	0.8	0.8	0.8
	Inverse Chi	0.89	0.8	0.84
0.5	Df-Model	0.875	0.7	0.78
	Df-Regpath	0.8	0.8	0.8
	Df-Cvpath	1	0.7	0.83
	Df-lambda	0.4	0.4	0.4
	sumz	0.7	0.9	0.8
	Inverse Chi	0.8	1	0.9
0.7	Df-Model	0.73	0.8	0.76
	Df-Regpath	0.89	0.8	0.84
	Df-Cvpath	1	0.6	0.77
	Df-lambda	0.66	0.6	0.63
	sumz	0.8	0.8	0.8
	Inverse Chi	0.9	0.9	0.95

### Computational efficiency

The time required to compute our proposed measures is calculated using an Intel(R) Core(TM) i7–5500U CPU@2.40 GHz processor on a dataset with varying dimension (i.e. no. of individuals (500, 750 & 1000) and markers (2000, 5000 & 10000) with all possible combination). Results of same is presented below in Table 3.

**Table 3:** Time required for running the datasets of varying combination of dimension using our proposed approach.

No. of individuals (n)	No. of markers (p)	Time (Minutes)
500	2000	305
	5000	560
	10000	965
750	2000	638
	5000	1367
	10000	2191
1000	2000	1182
	5000	2292
	10000	2570

In order to understand the effect of outlier on the genomic prediction accuracy, we have studied their effects on real dataset. First of all we have fitted LASSO regression with original experimental data say it as LASSO*. Then using the approach given by Rajaratnam *et al*.^17^, we have calculated p-values for all the four measures i.e. df-model, df-lambda, df-regpath, df-cvpath followed by combining these p-values into single value for each observation/genotype. Using the same we have identified the outlier in the response. The outlier and their corresponding marker genotype were dropped from the model and again LASSO is refitted using the modified data. In order to check robustness of our proposed approach, we have also fitted some of most commonly used methods for genomic selection i.e. Ridge Regression, Best Linear Unbiased Prediction (BLUP), Genomic-BLUP (GBLUP) and Bayesian methods. BLUP i.e. Best Linear Unbiased Prediction introduced by Henderson^33^ is used in a linear mixed model for prediction of random effects. GBLUP is an improved version of BLUP where additive genomic relationship matrix (G) is used as a variance-covariance matrix of random effect in the model^34^. For performance evaluations of methods under study, cross validation techniques is used. Data is divided into two parts i.e. training and testing sets such that training set comprises of 70% data and testing set of 30%. Former one is used for model building and later one for model evaluation. The performance of methods was evaluated by calculating prediction accuracy and prediction error. Whole procedures is repeated 100 times and prediction accuracy and prediction error is averaged and their respective standard error is calculated. Results of the same has been discussed below. Here Tables 4–9 reports the average prediction accuracy and prediction error (i.e. MSE) with their sampling variability (SE i.e. standard error) of the methods under study for dataset 1–6. In order to calculate gain in prediction accuracy all the fitted model were compared to baseline model i.e. LASSO and percentage change in prediction accuracy is calculated. In same way percentage reduction in MSE is also calculated.

**Table 4:** Mean and standard error of prediction accuracy and prediction error for various methods using dataset 1.

Methods	Accuracy	MSE	Accuracy SE	MSE SE	Percentage(%) gain in Accuracy	Percentage(%) reduction in MSE
LASSO*	0.44	0.82	0.06	0.08	NA	NA
Df-Model	0.47	0.83	0.06	0.09	6.8	0
Df-Regpath	0.55	0.60	0.05	0.07	25	27
Df-Cvpath	0.57	0.58	0.06	0.07	29.5	29.3
Df-Lambda	0.56	0.66	0.06	0.09	27.3	19.5
Inverse Chi	0.62	0.52	0.05	0.06	41	36.6
Logit	0.60	0.53	0.04	0.05	36.3	35.4
Meanp	0.59	0.56	0.06	0.05	34	31.7
Sumz	0.59	0.54	0.05	0.05	34	34.2
Regression with t-error	0.47	1.21	0.06	0.06	6.8	0
RR	0.56	0.60	0.05	0.06	27.3	27
GBLUP	0.60	0.81	0.05	0.06	36.3	0
Bayesian LASSO	0.57	0.61	0.06	0.07	29.5	25.6

**Table 5:** Mean and standard error of prediction accuracy and prediction error for various methods using dataset 2.

Methods	Accuracy	MSE	Accuracy SE	MSE SE	Percentage(%) gain in Accuracy	Percentage(%) reduction in MSE
LASSO*	0.26	0.96	0.09	0.14	NA	NA
Df-Model	0.36	0.96	0.11	0.16	38.5	0
Df-Regpath	0.28	1.01	0.10	0.14	7.7	0
Df-Cvpath	0.30	0.99	0.09	0.12	15.4	0
Df-Lambda	0.38	0.96	0.11	0.16	46.2	0
Inverse Chi	0.43	0.69	0.08	0.10	66	28.1
Logit	0.40	0.70	0.10	0.11	53.8	28.2
Meanp	0.34	0.83	0.08	0.14	30.8	13.5
Sumz	0.44	0.70	0.09	0.13	69	28
Regression with t-error	0.36	7.2	0.09	0.10	38.5	0
RR	0.46	0.72	0.08	0.11	77	26
GBLUP	0.48	0.71	0.07	0.11	84.6	26
Bayesian LASSO	0.45	0.68	0.09	0.10	73.1	29.2

**Table 6:** Mean and standard error of prediction accuracy and prediction error for various methods using dataset 3.

Methods	Accuracy	MSE	Accuracy SE	MSE SE	Percentage(%) gain in Accuracy	Percentage(%) reduction in MSE
LASSO*	0.52	13.3	0.1	2.7	NA	NA
Df-Model	0.60	9.5	0.09	2.2	15.4	28.6
Df-Regpath	0.57	10.8	0.08	1.9	9.6	18.8
Df-Cvpath	0.60	9.4	0.08	2.1	15.4	29.3
Df-Lambda	0.58	10.7	0.08	2.2	11.5	19.5
Inverse Chi	0.68	7.2	0.08	1.4	30.8	45.8
Logit	0.67	7.5	0.08	1.5	28.8	43.6
Meanp	0.66	7.9	0.08	1.5	26.9	40.6
Sumz	0.68	7.3	0.07	1.6	30.8	45.1
Regression with t-error	0.61	7.4	0.08	1.8	17.3	44.4
RR	0.68	7.5	0.07	1.3	30.8	43.6
GBLUP	0.65	7.5	0.07	1.5	25	43.6
Bayesian LASSO	0.62	8.9	0.8	1.6	19.2	33.1

**Table 7:** Mean and standard error of prediction accuracy and prediction error for various methods using dataset 4.

Methods	Accuracy	MSE	Accuracy SE	MSE SE	Percentage(%) gain in Accuracy	Percentage(%) reduction in MSE
LASSO*	0.47	21.6	0.09	4.5	NA	NA
Df-Model	0.47	20.8	0.10	3.9	0	6.3
Df-Regpath	0.59	15.8	0.07	3.1	25.5	28.8
Df-Cvpath	0.58	16.4	0.07	3.4	23.4	26.1
Df-Lambda	0.55	18.2	0.07	3.9	17	18
Inverse Chi	0.74	9.2	0.06	1.8	57.5	57.2
Logit	0.73	9.9	0.07	2.2	55.3	55.4
Meanp	0.70	11.2	0.07	2.5	48.9	49.5
Sumz	0.72	10.6	0.06	2.3	53.2	52.3
Regression with t-error	0.45	23.2	0.10	4.5	0	0
RR	0.70	10.9	0.06	2.5	48.9	49.5
GBLUP	0.64	21.7	0.08	5.3	36.2	2.3
Bayesian LASSO	0.62	13.5	0.08	2.7	31.9	39.2

**Table 8:** Mean and standard error of prediction accuracy and prediction error for various methods using dataset 5.

Methods	Accuracy	MSE	Accuracy SE	MSE SE	Percentage(%) gain in Accuracy	Percentage(%) reduction in MSE
LASSO*	0.38	0.37	0.10	0.06	NA	NA
Df-Model	0.44	0.33	0.09	0.06	13.6	10.8
Df-Regpath	0.42	0.35	0.09	0.08	9.1	5.4
Df-Cvpath	0.40	0.35	0.09	0.07	4.5	5.4
Df-Lambda	0.44	0.33	0.09	0.08	13.6	10.8
Inverse Chi	0.54	0.22	0.08	0.03	36.4	40.5
Logit	0.54	0.22	0.08	0.03	36.4	40.5
Meanp	0.52	0.25	0.08	0.03	31.8	32.4
Sumz	0.54	0.22	0.08	0.03	36.4	40.5
Regression with t-error	0.34	0.38	0.10	0.06	0	0
RR	0.53	0.22	0.08	0.10	39.5	40.5
GBLUP	0.57	0.35	0.09	0.10	50	5.4
Bayesian LASSO	0.55	0.30	0.10	0.10	44.73	18.9

**Table 9:** Mean and standard error of prediction accuracy and prediction error for various methods using dataset 6.

Methods	Accuracy	MSE	Accuracy SE	MSE SE	Percentage(%) gain in Accuracy	Percentage(%) reduction in MSE
LASSO*	0.45	0.07	0.10	0.02	NA	NA
Df-Model	0.49	0.06	0.10	0.02	8.9	14.3
Df-Regpath	0.47	0.06	0.10	0.02	4.5	14.3
Df-Cvpath	0.49	0.06	0.10	0.02	8.9	14.3
Df-Lambda	0.51	0.05	0.09	0.009	13.4	28.6
Inverse Chi	0.55	0.04	0.07	0.006	22.2	42.9
Logit	0.54	0.04	0.07	0.007	20	42.9
Meanp	0.57	0.04	0.08	0.007	26.67	42.9
Sumz	0.54	0.04	0.08	0.007	20	42.9
Regression with t-error	0.44	0.22	0.10	0.10	0	0
RR	0.52	0.04	0.08	0.01	15.5	42.9
GBLUP	0.50	0.06	0.09	0.02	11	14.3
Bayesian LASSO	0.42	0.07	0.10	0.01	0	0

Here all the analysis has been carried out using R (R Development Core Team 2019). LASSO model is fitted using R package glmnet^35^, other methods like BLUP, GBLUP are fitted using rrBLUP package^36^ with mixed.solve and kin.blup function respectively. Ridge regression is fitted using Gustavo de los Campos R code, fitting this require heritability of underlying trait. For better description, heritability for each traits under study is provided in the supplementary material (Table S1). Regression with t-error fitted using R package “hett” (using tlm function)^37^. Degree of freedom is estimated for different dataset used in study by using the tlm function with option (estDof = TRUE), available in R package “hett” and then t-regression is fitted.

In this Table 4 and others (Tables 5–9) LASSO* represents LASSO regression fitted in original data (i.e. without any treatment to possible outlier), next four methods in the table represent performance of LASSO in the absence of outlier (i.e. possible outlier and corresponding genotype marker genotype dropped from the model detected using LASSO diagnostic) whereas next four methods in the table represent performance of LASSO in the absence of outlier (i.e. possible outlier and their corresponding marker genotype to be dropped from original data detected by our various p-value based meta-analysis approach). Last four methods shows the performance of other methods on our proposed approach.

In order to assess gain in the prediction accuracy for different datasets under study, It could be observed that there is significant amount of gain in prediction accuracy (Tables 4–9) as compare to their counterparts (41% increase in case of dataset 1, 69% for dataset2, 31% for dataset 3, 57% for dataset 4, 36% for dataset 5 and 27% for dataset 6). In case of Prediction error it can observed from results (Tables 4–9) that MSE for our proposed approach has been significantly reduced (i.e. 37% for dataset 1, 28% for dataset 2, 46% for dataset 3, 57% for dataset 4, 40% for dataset 5 and 43% for dataset 6). It shows clear advantage of our integrated approach (i.e. p-value based meta-analysis method) over the existing approach. In order to see that gain in terms of predictions performance is not only restricted to LASSO, we have also investigated the performance of integrated approach by using most commonly used GS models (RR, GBLUP etc.). It can be marked with confidence that gain in terms of prediction performance has been maintained to other methods also (Tables 4–9).

**Figure 2:**
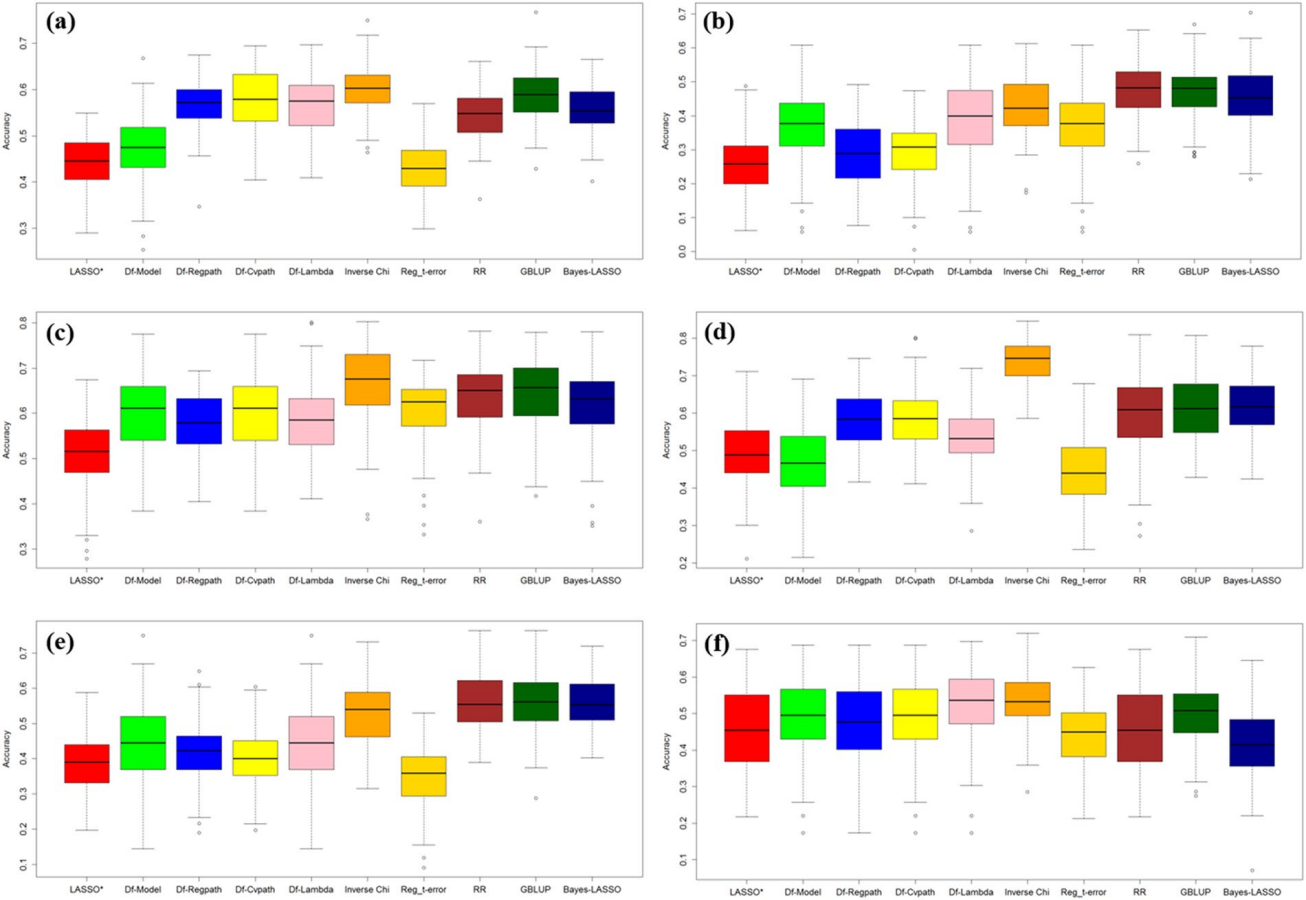
Box plot of prediction accuracy for different methods under study using various datasets (**a**) dataset 1 (**b**) dataset 2 (**c**) dataset 3 (**d**) dataset 4 (**e**) dataset 5 (**f**) dataset 6.

In Fig. 2, each graph (Fig. 2a–f) contains the ten box plot for prediction accuracy for dataset 1–6 respectively. In each figure first box plot shows the prediction accuracy by fitting simple LASSO regression, next four box plot shows the prediction accuracy calculated following the approach of Rajaratnam *et al*.^17^ and next method (Inverse Chi) represent performance of LASSO in the absence of outlier (i.e. possible outlier and their corresponding marker genotype to be dropped from original data detected by p-value based meta-analysis approach i.e. Inverse Chi). Last four methods shows the performance of other GS methods on our proposed approach. These Box plots shows the distribution of prediction accuracy with their SE, estimated over 100 replications.

**Figure 3:**
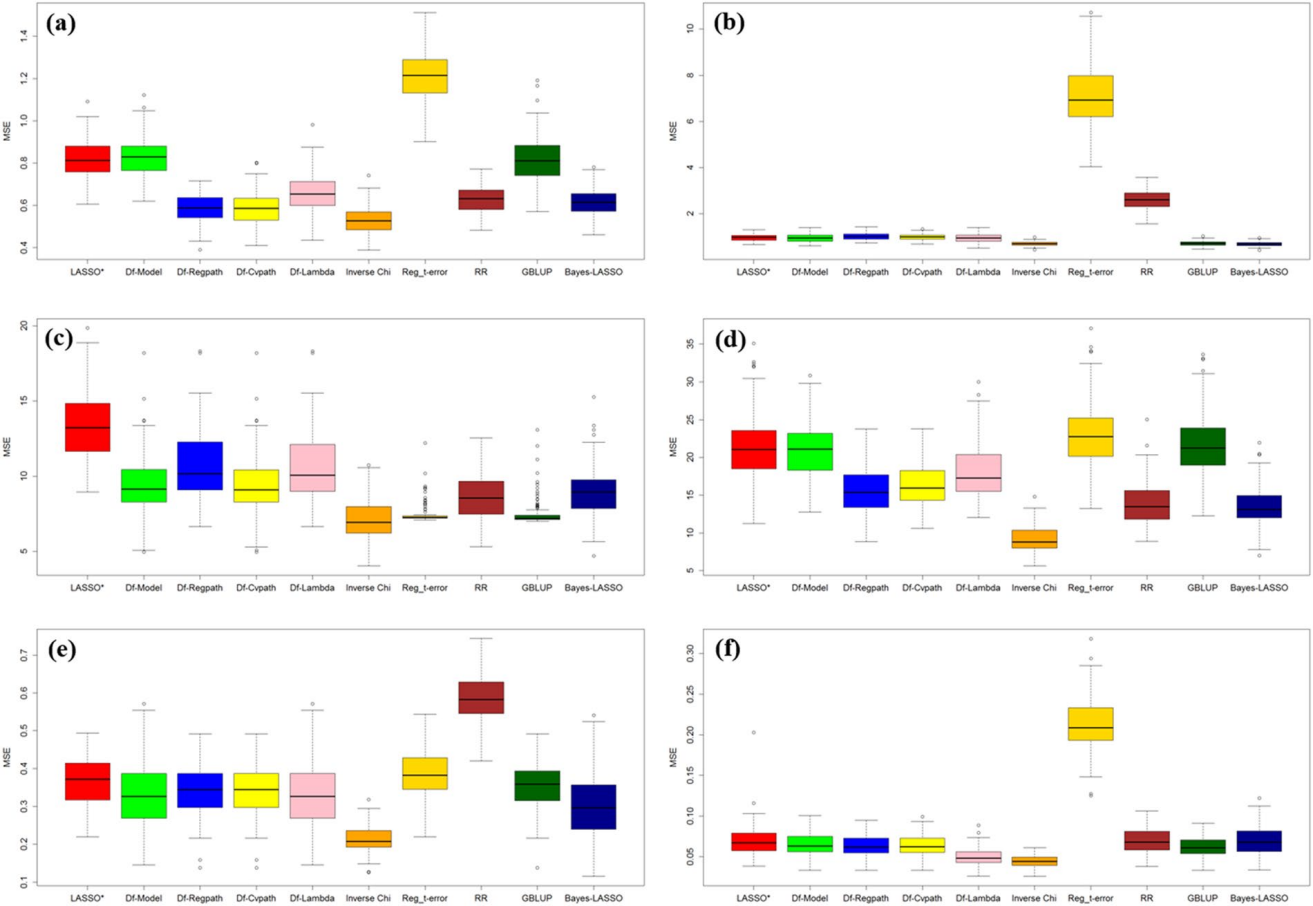
Box plot of prediction error (MSE) for different methods under study using various datasets (**a**) dataset 1 (**b**) dataset 2 (**c**) dataset 3 (**d**) dataset 4 (**e**) dataset 5 (**f**) dataset 6.

In Fig. 3, each graph (Fig. 3a–f) represents ten box plot for prediction error for dataset 1–6 on the same pattern of boxplot to Fig. 2. These boxplots represents the distribution of the MSE values over 100 runs. These plots (Figs. 2 and 3) show a clear cut advantage of our proposed approach over the LASSO diagnostic given by Rajaratnam *et al*.^17^, in improving genomic prediction accuracy and other existing approach. In almost every scenario i.e. wheat and maize dataset (dataset 1–6), prediction accuracy has been improved and prediction error get minimized. Clear distinctions of estimated accuracy and prediction error shows the importance of outlier detection for estimating more accurate GEBVs leads to enhanced prediction accuracy. It can be summarized from the Tables 4–9 that among p-value combination methods Inverse Chi, logit and sumz performed equally although advantage goes to Inverse Chi and sumz over logit and meanp method.

Ranking of the various methods used for performance evaluation has been done using multi criteria based decision method called TOPSIS. Result of same has been given in Tables S2 and S3 (Supplementary Information). It can be concluded from Tables S2 and S3 that our integrated approach (based on p-value meta-analysis) using Inverse Chi method ranked first among other p-value based meta-analysis methods (i.e. logit, meanp and sumz) for both in case of dataset 1 and dataset 2 and same pattern has been observed for other datasets also using TOPSIS methods based on multi criteria.

### Mean shift as substitute for deletion

Instead for deleting the observation flagged as outlier here we have substituted the outlier with the mean shift of data using mean shift outlier model (MSOM)^38^. Here one or more observation is assumed to be introduced from a shifted location as compare to remaining observation. This method can be important for robust modelling where we identify the observation flagged as outlier with separate mean shift effect instead of dropping them from model. Earlier we have fitted the model to real and simulated data and for each observation outliers are identified (p-value < 0.05) based on p-value combination approach. Here instead of deleting the observation flagged as outlier, we have replaced them with separate mean shift effect (using MSOM).

Best linear unbiased prediction i.e. BLUP^33^ and GBLUP^34^ model is fitted on original data and data where outliers are treated with MSOM. A Significant improvement in the accuracy over baseline model (using original data as such) has been observed. Details of same is presented in Table 10.

**Table 10:** Effect of Mean Shift Model over baseline model on accuracy of the genomic prediction using BLUP.

Accuracy/Data	Dataset 1	Dataset 2	Dataset 3	Dataset 4	Dataset 5	Dataset 6
Original Data fitted using BLUP	0.50	0.42	0.60	0.62	0.45	0.40
Mean Shift fitted using BLUP	0.97	0.68	0.93	0.81	0.85	0.82
Mean Shift fitted using GBLUP	0.98	0.77	0.95	0.88	0.94	0.86

## Conclusion

Impact of outlier on genomic prediction accuracy has been explored. In this study, a new efficient method using meta-analysis for outlier detection in genomic data has been proposed. It has been shown that by implementing efficient diagnostic measure for outlier detection, accuracy of GS model can be improved. Comparative study has been made among various existing methods of outlier detection in high dimensional genomic data for their impact on accuracy of genomic estimated breeding value. It has been observed that our proposed method outperformed among existing methods.

## Supplementary information

Supplementary Information.

## Data Availability

All secondary datasets used in this study are publicly available.
