# Supplementary material for: Statistical Approach for Improving Genomic Prediction Accuracy through Efficient Diagnostic Measure of Influential Observation
Source: Sci Rep. 2020 May 21;10:8408. doi: 10.1038/s41598-020-65323-3 (PMC7242349; doi:10.1038/s41598-020-65323-3)
Supplement: Supplementary file 1 — Supplementary Information. [file 41598_2020_65323_MOESM1_ESM.pdf]

Statistical Approach for Improving Genomic Prediction Accuracy through Efficient Diagnostic Measure of Influential Observation

Neeraj Budhlakoti[1], Anil Rai[1], D C Mishra[1*]

[1]Centre for Agricultural Bioinformatics, ICAR-Indian Agricultural Statistics Research Institute, New Delhi, 110012

*Corresponding author (dwij.mishra@gmail.com)

Supplementary Information

Table S1: Narrow sense heritability (h^2) of different traits under study

Table S2: Ranking of different methods based on their performance against multiple criteria using TOPSIS (using dataset 1)

Table S3: Ranking of different methods based on their performance against multiple criteria using TOPSIS (using dataset 2)

Table S1: Narrow sense heritability (h^2) of different traits under study

Dataset	Heritability (Narrow sense)
Dataset 1 : (Wheat, Trait: GY)	0.67
Dataset 2 : (Maize, Trait: GY)	0.46
Dataset 3 : (Wheat, Trait: DTH)	0.81
Dataset 4 : (Wheat, Trait: TKW)	0.73
Dataset 5 : (Wheat, Trait: $GY_{Irrigated}$)	0.87
Dataset 6 : (Wheat, Trait: $GY_{Draught}$)	0.84

Table S2: Ranking of different methods based on their performance against multiple criteria using TOPSIS (using dataset 1)

Methods	TOPSIS Score	Rank
**LASSO***	0.04417437	9
**Df-Model**	0.08690076	8

**Df-Regpath**	0.69172193	6
**Df-Cvpath**	0.75658872	5
**Df-Lambda**	0.56387915	7
**Inverse Chi**	0.94412063	1
**Logit**	0.93474351	2
**meanp**	0.83710633	4
**sumz**	0.88746934	3

Table S3: Ranking of different methods based on their performance against multiple criteria using TOPSIS (using dataset 2)

**Methods**	**TOPSIS Score**	**Rank**
**LASSO***	0.10463493	8
**Df-Model**	0.42201140	6
**Df-Regpath**	0.09677422	9
**Df-Cvpath**	0.19527985	7
**Df-Lambda**	0.48472555	5
**Inverse Chi**	0.95591532	1
**Logit**	0.81931136	3
**meanp**	0.48693328	4
**sumz**	0.94082110	2
